# Analysis of the circular RNA transcriptome in endometrial cancer

**DOI:** 10.18632/oncotarget.23534

**Published:** 2017-12-20

**Authors:** Bei Jun Chen, Frances L. Byrne, Konii Takenaka, Susan C. Modesitt, Ellen M. Olzomer, James D. Mills, Rhonda Farrell, Kyle L. Hoehn, Michael Janitz

**Affiliations:** ^1^ School of Biotechnology and Biomolecular Sciences, University of New South Wales, Sydney, NSW, Australia; ^2^ Division of Gynecologic Oncology, Obstetrics and Gynecology Department, University of Virginia Health System, Charlottesville, VA, USA; ^3^ Department of Pathology, Academic Medical Center, University of Amsterdam, Amsterdam, The Netherlands; ^4^ Gynecologic Oncology, Royal Hospital for Women, University of New South Wales, Sydney, NSW, Australia; ^5^ Paul-Flechsig-Institute for Brain Research, University of Leipzig, Leipzig, Germany

**Keywords:** circRNAs, RNA-Seq, transcriptome, endometrial cancer, hotspot genes

## Abstract

Circular RNAs (circRNAs) are a naturally occurring family of non-coding RNA that may regulate gene expression in mammals. circRNAs are more stable than messenger RNAs due to their resistance to RNA exonuclease. A growing body of evidence has shown that the expression of circRNAs is regulated during development in a tissue-specific manner. CircRNAs have been implicated in a number of cancers; however, their role in endometrial cancer (EC) is completely unknown. Here, we report the circular transcriptome specific for EC as determined by RNA sequencing. We found that the overall abundance of circRNAs is lower in EC than in normal endometrium. Further, there are numerous ‘hotspot’ genes from which circRNAs are transcribed that may account for alterations in circRNA expression between the normal and malignant endometrium. Most importantly, we have also identified circRNAs that are differentially expressed between malignant and normal endometrial tissue. The functional significance of these circRNAs in cancer remains to be determined, but they may serve as potential biomarkers for the diagnosis of EC or monitoring of EC progression.

## INTRODUCTION

Endometrial cancer (EC) is the fifth most common cancer in women, accounting for 4.8% of all malignancies and 2.1% of all cancer-related deaths in women [1]. EC is often classified into two subtypes, type I and II, based on clinical, pathological and molecular characteristics [2]. Type I cancers are typically low stage and grade endometrioid histology that are positive for the estrogen and progesterone receptors, and strongly associated with obesity [3]. In contrast, type II cancers are usually estrogen-independent, advanced grade and stage endometrioid or clear-cell/serous histology, are not typically linked to obesity, and have a worse prognosis [3].

More recent investigations using next-generation sequencing technologies have facilitated a new evolution for the molecular classification of endometrial cancer. The TCGA project defines patients into categories that correlate with prognosis by 4 molecular subgroups including: (1) POLE (ultramutated), (2) microsatellite instability hypermutated, (3) copy-number low (microsatellite stable), and (4) copy-number high (serous-like) [4, 5], while the Leiden/TransPORTEC and Vancouver/ProMisE molecular classification systems have different risk stratification parameters to define molecular subgroups associated with prognosis [6].

Historically regarded as the product of aberrant splicing, circular RNAs (circRNAs) are a recently identified endogenous RNA species formed by spliceosomal joining of the downstream 5′ splice site of an exon with the 3′ splice site of either the same or an upstream exon, in a process known as back-splicing [7–9]. Back-splicing takes place at the pre-mRNA stage and competes with canonical splicing [10]. CircRNAs are widely present in the eukaryotic tree of life and studies have shown that their expression is regulated in a development- and tissue-specific manner [11–14]. CircRNAs are resistant to cleavage by RNA exonuclease and have extended half-life time of up to 48 hours, as compared to 10 hours for linear RNAs, making them appealing biomarker candidates for cancer and neurodegenerative diseases [15–17].

The involvement of circRNAs in cancer pathology has recently become a subject of intensive research. For example, it has been demonstrated that in colorectal and ovarian cancer the abundance of circRNAs, measured by the ratio of circRNAs to linear isoforms, is lower in tumor samples [16]. Furthermore, this ratio correlated negatively with tumor cell proliferation rate. Another study reported the enrichment of circRNAs in exosomes produced by liver cancer cells [18]. In addition, exosomal circRNAs in peripheral blood of colon cancer patients have unique expression patterns as compared to those in healthy controls [18]. These initial studies indicate circRNAs may serve as potential biomarkers in cancer diagnosis and monitoring of cancer progression.

Despite increasing evidence that circRNAs have roles in oncogenesis and cancer progression, nothing is known about the role of these transcripts in EC pathology. In our previous study, we reported lincRNA profiles of type I EC tissue from six patients, paired with adjacent non-cancerous endometrial tissue (Table 1) [19]. Using the same RNA-Seq data sets, we applied a computational pipeline which detects and tests differential expression of circRNAs between malignant and normal endometrial tissue. To our knowledge, this is the first study of circular transcriptome profiling of EC where we describe the circular transcriptome landscape, characterize features of circular transcripts expressed in EC, and detect differentially expressed circRNAs that may serve as potential biomarkers in EC diagnosis and progression monitoring.

**Table 1:** Patient and tissue sample chart

Sample No.	Condition	Patient No.
1N	Normal	1
2T	EC	
3N	Normal	2
4T	EC	
5N	Normal	3
6T	EC	
7N	Normal	4
8T	EC	
9N	Normal	5
10T	EC	
11N	Normal	6
12T	EC	

## RESULTS

### Global expression of circRNAs in endometrial cancer

**Figure 1:**
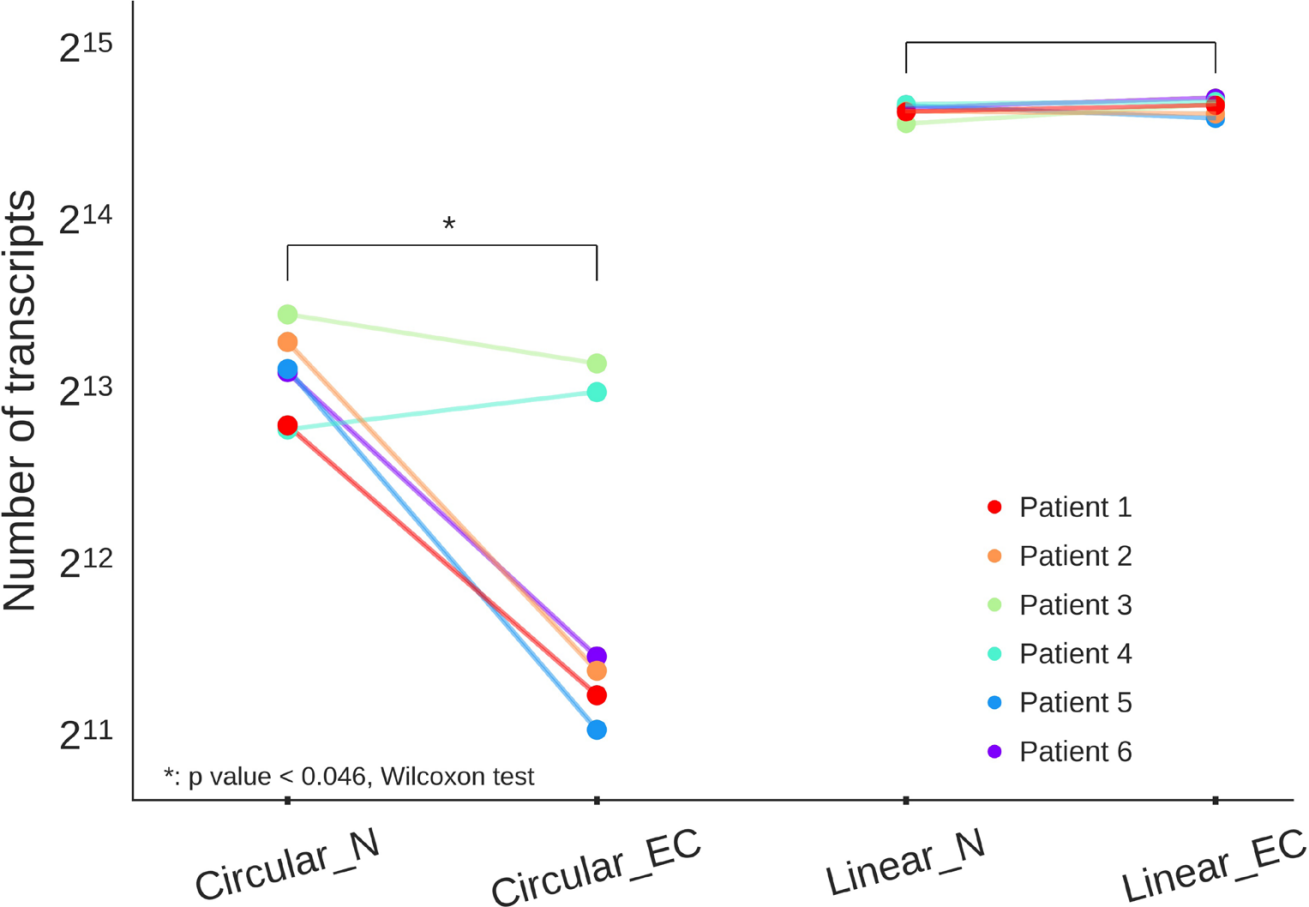
Total number of circular and linear transcripts in six endometrial cancer tissues (Circular_EC, Linear_EC) and six patient-matched normal tissues (Circular_N, Linear_N) shown as dots in six distinct colors, with lines connecting two samples from the same patient Overall there are less circular transcripts in EC samples than in normal tissues.

We estimated global expression of circRNAs in EC and matched normal endometrial tissue and detected a total of 25,154 non-redundant circRNAs expressed across the two groups: 21,340 in normal and 14,707 in EC tissue, respectively. As depicted in Table 2, 30.8% of all gene loci, detected as expressed in normal and EC samples, also produced circRNA transcripts. The ratio of circular to linear RNA isoforms was lower in EC (23.9%) compared to normal endometrial tissues (30.1%). There were significantly less circular transcripts in EC than in normal endometrium (*p* value < 0.046, Wilcoxon test). On the contrary, linear RNAs discovered from the same data, showed no difference in numbers of transcripts between EC and normal endometrium (Figure 1).

**Table 2:** Global metrics of expressed linear and circular RNAs in normal and EC tissue

Condition	Number of genes expressing linear RNAs^a^	Number of genes expressing circRNAs	% circRNA to linear RNAs
Normal	16,293	4,901	30.1%
EC	16,188	3,875	23.9%
Combined^b^	16,674	5,137	30.8%

^a^: CPM ≥ 1; ^b^: Non-redundant set.

### CircRNA sequence composition

**Figure 2:**
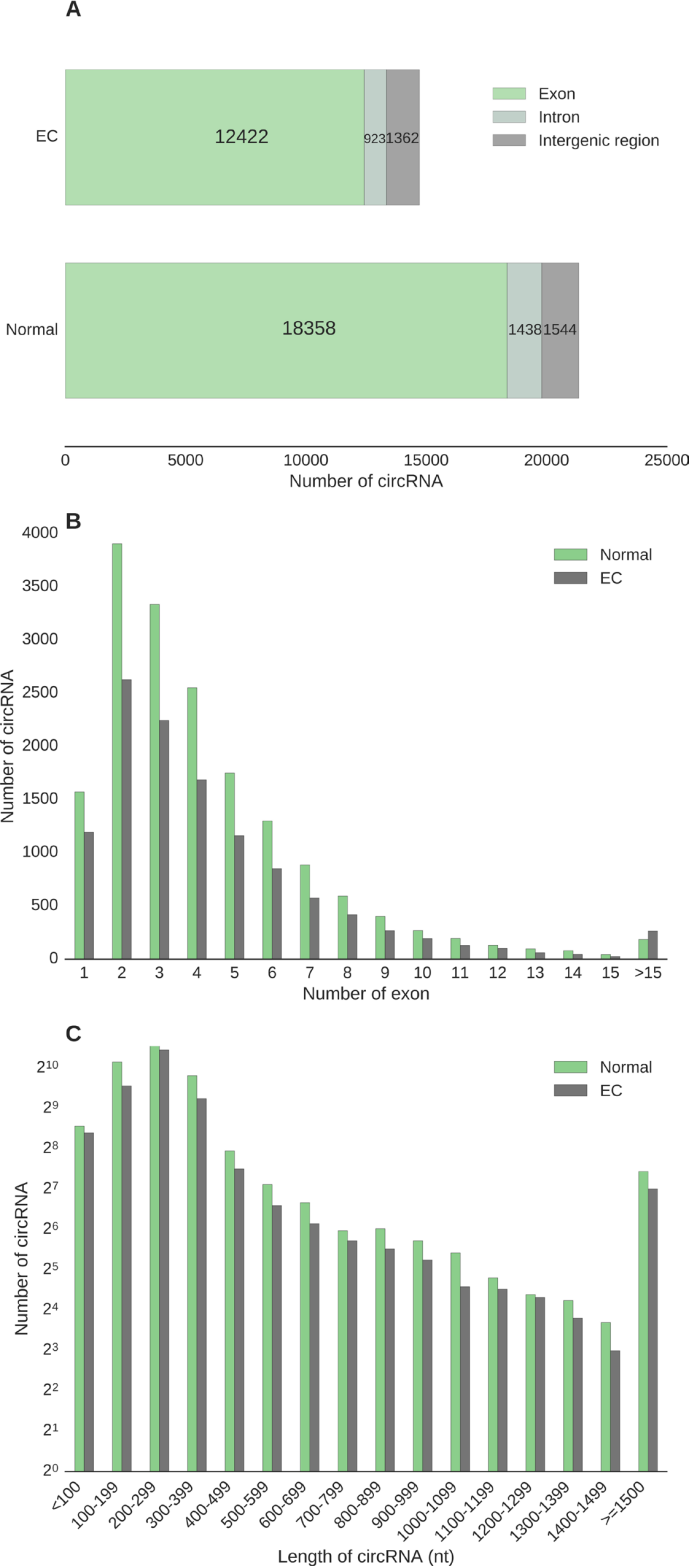
Characteristics of genomic structure and location of circular RNAs expressed in endometrial cancer and their adjacent normal endometrial tissues (**A**) Distribution of transcript genomic position. (**B**) Distribution of exon composition. (**C**) Distribution of transcript length of single and two-exon circular RNAs.

Individual circRNAs were defined as expressed when at least one back-spliced read per individual circRNA transcript was detected. The majority of circRNAs discovered were transcribed from exons of known genes, accounting for 86% circRNAs found in normal samples and 84.5% in EC (Figure 2A). A small proportion of circRNAs aligned to introns (6.7% in normal and 6.3% in EC) and the intergenic portion of the genome (7.2 % in normal and 9.2% in EC) (Figure 2A).

Circular RNAs transcribed from 2 exons (two-exon circRNAs) were the most abundant circRNAs in both normal (22.6%) and EC (22.2%) tissues, followed by three-exon circRNAs (19.3% in normal and 18.9% in EC) (Figure 2B). Figure 2B illustrates distribution of sequence composition for detected circRNAs, of which the number of exons in circRNA peaks at two-exon in both normal endometrial and EC tissues.

We next examined the length of single and two-exon circRNAs given that splicing variation is limited in these transcripts. Most circRNAs ranged from 100 to 400 nucleotide (nt) in both normal endometrium and EC tissues (Figure 2C), with a few transcripts stretched to 8 kilo nt (normal) or 11 kilo nt (EC), despite only having at most two exons in each transcript. This size range is in agreement with previous genome-wide surveys of the circular transcriptome in human brain, liver cancer, and fetal samples [17, 18, 20]. The median spliced length of circRNAs in the 100–400 nt range was 239 and 240 nt for normal endometrium and EC tissues, respectively (Figure 2C). There were no significant differences in length distribution between circRNAs detected in normal endometrium and EC tissues (*p* value > 0.99, Kolmogorov-Smirnov test).

### circRNA hotspot genes

**Figure 3:**
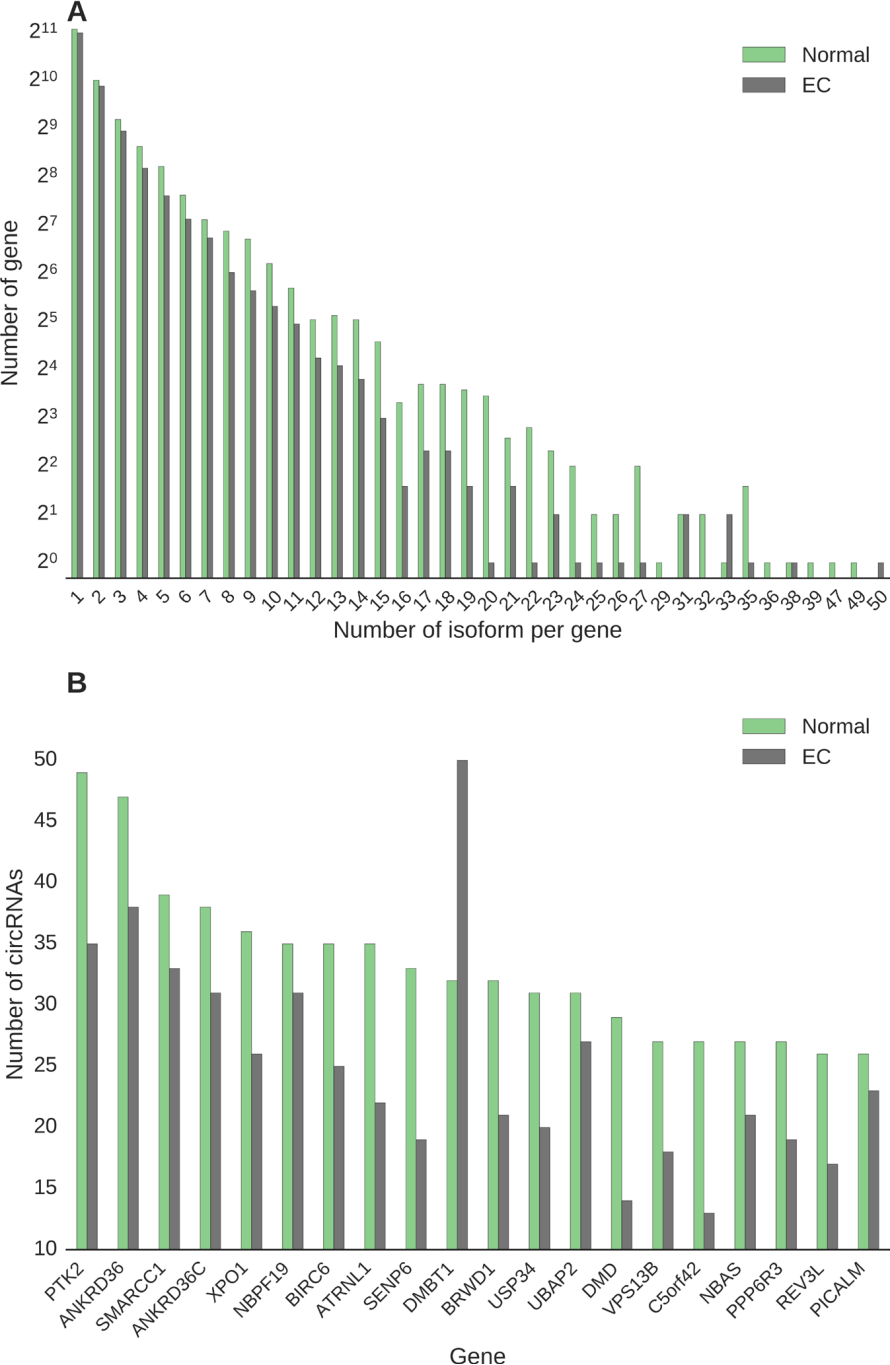
(**A**) Number of circular isoforms expressed by hotspot genes, which are genes that express more than ten circRNA isoforms, in endometrial cancer and normal tissues. (**B**) Expression of the 20 hotspot genes that expressed at least 25 circular isoforms in normal tissue, together with their expressions in endometrial tissues.

Next, we estimated the number of unique circular transcripts per gene in EC and normal endometrial tissue samples (Figure 3). Most of the genes expressed a single circRNA isoform, independently of tissue sample type (Figure 3A). We identified 280 genes in the normal endometrial tissue transcriptome, and 123 in the EC tissue, which produced over ten circRNAs and thus represent ‘circRNA hotspots’ (Supplementary Table 1). Table 3 lists the top ten unique hotspot genes expressed in normal (N) tissue and the eight unique hotspots genes expressed in endometrial cancer (EC) tissue. For the 280 hotspots expressed in normal endometrial tissue, we assessed whether the number of circRNAs produced by the same gene were different in EC tissue and observed a significant decrease in isoform numbers in EC tissue (*p* < 9.64 × 10^–42^, Wilcoxon test). As depicted in Figure 3B, there is a general decrease of isoform variety in EC. The greatest decrease in isoform numbers was observed for the Dystrophin gene (*DMD*) with 29 specific circRNA isoforms expressed in normal endometrial tissue and 14 in EC tissue (Figure 3B). In contrast, the number of circRNA isoforms expressed by the Deleted In Malignant Brain Tumors 1 gene (*DMBT1*) increased from 32 in normal endometrium to 50 in EC (Figure 3B).

**Table 3:** Top ten unique hotspot genes expressed in normal (*N*) tissue and the eight unique hotspots genes expressed in endometrial cancer (EC) tissue

Gene	No. of circular transcripts		If any circular transcript is differentially expressed	If linear transcript is differentially expressed
	*N*	EC		
*NOX4*	**23**	10	yes	yes
*PTPN4*	**20**	10	no	no
*ST7L*	**19**	10	no	no
*KCNMA1*	**19**	10	no	yes
*RUFY2*	**18**	9	no	no
*RALGAPA1*	**18**	10	no	no
*PDLIM5*	**18**	10	yes	yes
*EPS15*	**18**	9	no	no
*CSPP1*	**18**	7	no	no
*ZMYM4*	**17**	10	no	no
*DNAH14*	3	**18**	yes	yes
*MT-RNR2*	8	**12**	no	no
*RABGAP1*	10	**12**	no	no
*ESR1*	9	**11**	no	no
*FIP1L1*	10	**11**	no	yes
*GFPT1*	9	**11**	no	no
*INADL*	9	**11**	no	yes
*PCNX*	10	**11**	no	no

### Correlations between circRNAs and microRNAs expression in endometrial cancer

*ZNF91* mRNA has numerous target sites for miR-23, miR-181, and miR-199 [21]. Guo and colleagues identified 24 binding sites for miR-23 and seven for miR-199 in one of the circular ZNF91 (circ-ZNF91) transcripts [22]. Following this previous finding, we examined the correlation between expression of circ-ZNF91 with those of miR-23 and miR-199, respectively (Supplementary Figure 1). The Pearson’s *r* was –0.37 for correlation between circ-ZNF91 and miR23B, and –0.45 for circ-ZNF91 and miR199A2 expression, showing a moderate negative correlation in both cases (Supplementary Figure 2).

### circRNAs differentially expressed in EC

Pairwise comparison between matched normal endometrial tissue and EC tissue from six patients discovered 120 circRNAs that are differentially expressed (*p* value < 0.01, likelihood ratio test). Supplementary Table 2 provides genomic details of all differentially expressed circRNAs and their expression metrics in all 12 samples. Out of the 120 circRNAs, 103 circRNAs were transcribed from the exons and 3 from introns of known genes, while the remaining 14 circRNAs were expressed from intergenic loci. Of the 120 differentially expressed circRNAs, 22 were up-regulated and 98 were down-regulated in EC (Supplementary Table 2). Of the 120 differentially expressed circRNAs, 75 (Supplementary Table 2, highlighted in yellow) had uniform patterns of expression between EC and normal endometrial tissue i.e. the expression of these circRNAs were either all up-regulated in EC tissue or all down-regulated in EC tissue, compared with normal endometrium. The most significantly altered circRNAs that show uniform direction of expression in all six patients are listed in Table 4.

**Table 4:** Two up-regulated and the top ten down-regulated circRNAs that show unified direction of expression in all six endometrial cancer (EC) samples

Gene	Patient 1		Patient 2		Patient 3		Patient 4		Patient 5		Patient 6		Fold change	*p* value
	*N*	EC	*N*	EC	*N*	EC	*N*	EC	*N*	EC	*N*	EC		
chr16:21457771| 21889392^*^	0.53	0.93	0	0.28	0	0.75	0	0.79	0	0.87	0.94	1.13	5.23	2.64E-07
*DNAH14*	0.07	0.16	0.04	0.07	0.05	0.13	0	0.21	0.09	0.37	0	0.46	3.65	8.56E-09
*FNDC3A*	0.16	0	0.21	0	0.10	0	0.16	0.04	0.20	0	0.17	0	–3.50	5.92E-04
*TBC1D1*	0.14	0	0.19	0	0.13	0	0.18	0.06	0.24	0	0.15	0	–3.40	1.38E-03
*ADGRD1*	0.21	0	0.04	0	0.18	0.04	0.07	0	0.26	0	0.07	0	–3.36	1.30E-03
*KCTD3*	0.09	0	0.06	0	0.20	0	0.09	0.04	0.20	0	0.15	0	–3.36	3.96E-03
*APLF*	0.23	0	0.15	0	0.15	0.11	0.21	0	0.15	0	0.10	0	–3.26	3.94E-03
*ALS2*	0.25	0	0.15	0	0.15	0	0.21	0.19	0.20	0	0.10	0	–3.26	8.78E-03
*ERI3*	0.11	0	0.04	0	0.13	0	0.23	0.06	0.24	0	0.07	0	–3.20	3.52E-03
*ATP2C1*	0.07	0	0.13	0	0.08	0	0.23	0.04	0.09	0	0.10	0	–3.16	2.63E-03
*GPHN*	0.07	0	0.10	0	0.18	0.13	0.27	0	0.13	0	0.15	0	–3.13	7.23E-03
*VPS13A*	0.09	0	0.10	0	0.05	0	0.21	0.09	0.20	0	0.37	0	–3.11	4.15E-03

^*^: represents the back-splice site.

## DISCUSSION

To our knowledge, this is the first study to report the circular RNA transcriptome of EC. Through our analyses we have discovered that: 1) the overall abundance of circRNAs is lower in EC than that of the normal endometrium; 2) most circRNAs are transcribed from exons in the endometrium; and 3) there are numerous ‘hotspot’ genes from which circRNAs are transcribed that may account for alterations in circRNA expression between the normal and malignant endometrium. Importantly, we have also identified circRNAs that are differentially expressed between malignant and normal endometrial tissue that may one day serve as potential biomarkers for the diagnosis of EC or monitoring of EC progression.

Two intronic circRNAs, HSPG2 and RP11-255H23.4, were only expressed in the normal tissue, while their mRNAs transcribed from the parent genes were both increased in EC tissues. *HSPG2* encodes the perlecan protein which is a key heparin sulphate proteoglycan of the basement membrane (BM). HSPG2 binds to a number of growth factors via its heparin sulphate glycosaminoglycan (HS-GAG) chains and regulates endothelial growth and re-generation [23]. Interestingly, decreased HS-GAG expression of BM was associated with tumor progression in EC [23]. In contrast to exonic circRNAs with well-established miRNA sponge function, Zhang *et al.* showed that circular intronic RNAs (ciRNAs) have little enrichment for miRNA target sites. Further, knock-down of ciRNAs resulted in decreased expression of their parent genes in human cells, implying a cis-regulatory role of circRNAs on their parent genes [24]. In the present study, however, the absence of the two circRNAs in EC was accompanied by the increased expressions of their parent genes, suggesting a different role of circular HSPG2 and RP11-255H23.4 in EC pathology.

MicroRNAs (miRNAs) are another non-coding RNA species transcribed from short hairpin precursors [25]. They participate in fine-tuning of gene expression and deregulation of miRNA may lead to cascades of cellular events that ultimately contribute to tumorigenesis [26–30]. Most circRNAs with a well-established role in cancer exert their effects via the circRNA-miRNA-mRNA axis, by acting as miRNA sponges [9, 31–35]. Circular ZNF91 has 24 binding sites for miR-23 and seven for miR-199 [22]. In the present study circular ZNF91 expression was inversely correlated to that of miR23B and miR199A2. Both miRNAs have been shown to be involved in a variety of human cancers. For example, miR23B targets a mitochondrial tumor suppressor proline oxidase (*POX*) and over-expression of miR23B results in down-regulation of *POX* in renal cancer [36]; in prostate cancer miR23B leads to attenuated *Sre* expression and decrease in tumor growth in nude mice; miR23B also exhibited metastasis suppression effect in colon cancer and its targets include *FZD7* or *MAP3K1* [37]. Taken together, miR23B plays complex roles in cancer pathways and could be either oncogenic or tumor suppressive in different cancers. In the case of miR122A2, a study published in 2010 showed that it forms a unique cluster with miR214 in epithelial ovarian cancer cells (Type II/CD44-) that was responsible for the regulation of the pro-inflammatory NF-kB pathway and the AKT survival pathway [38]. In the present study, the inverse correlation between circular ZNF91 with miR23B and miR122A2 may be a result of circ-ZNF91 acting as miRNA sponge, tethering away miR23B and miR122A2 in the cell.

Hotspot genes have been defined as producing more than ten distinct circRNA isoforms in a given tissue or cell [39] and a number of hotspot genes expressing multiple circular isoforms has been described previously [17, 40]. The current view is that two splicing mechanisms, carried out by two separate sets of transcription factors, may regulate linear and circular RNA alternative splicing independently in the cell [40, 41]. In the present study we identified a total of 288 (165 unique in normal tissue, 8 unique in EC, and 115 common to both conditions) hotspot genes. Out of 280 identified hotspot genes in the normal transcriptome, 165 ceased to produce more than ten circular transcripts after cancerous transformation therefore were unique to the normal transcriptome. To the contrary, only 123 hotspot genes were expressed after cancerous transformation: of which 8 were specific to cancer while the remaining 115 were also hotspots in the normal tissue. Taken together, our data shows that decreased overall abundance of circRNAs in EC was accompanied by the decrease of circular isoform diversity.

Out of the 8 unique hotspot genes (defined by more than 10 isoforms) in EC, estrogen receptor 1 (*ESR1*) gene expressed 9 circular isoforms in normal tissue and 11 in EC, with insignificant changes in both circular and linear RNA abundance across two conditions. Although estrogen receptor 1 plays an important role in hormonal responses in estrogen-sensitive tissues, studies demonstrated that its cellular abundance has little significance in prognostic relevance of endometrial cancer [42, 43]. Our study provides another direction in the clinical use of *ESR1* gene: the presence of cancer-specific ESR1 circular isoforms, instead of estrogen receptor 1 protein levels or transcript abundance, might be a potential biomarker for EC diagnosis and progression.

*DNAH14*, which encodes a heavy chain of the dynein motor protein, is another unique hotspot gene in EC and also one of the 120 differentially expressed circRNAs in EC. *DNAH14* generated three circular isoforms in normal endometrial tissues and 18 circular isoforms in EC tissue. The increase of *DNAH14* isoform number was accompanied by the up-regulation of both circular and linear transcript expression in EC tissue. Interestingly, a recent study has identified *DNAH14* as one of the 21 passenger genes in EC suggesting that *DNAH14* aberration could interfere with cancer-related pathways [44].

In commonly expressed hotspot genes in normal and EC tissue, we noticed two genes that underwent significant changes in circular transcript composition. The number of unique circular isoforms expressed by the *DMD* gene decreased from 29 in normal tissue to 14 in EC tissue; and the *DMBT1* gene increased from 32 in normal tissue to 50 in EC tissue. In normal human skeletal muscle, a specific circRNA is formed by the back-splicing of exon 55–45 of *DMD* locus. It has been proposed that this circRNA formation is a result of multi-exon skipping during *DMD*-specific splicing [45]. DMBT1 levels are elevated in biliary intraepithelial neoplasia and its absence in biliary tract of cancer patients correlates with poorer survival, thus suggesting suppressive effect *DMBT1* expression on tumor growth [46]. Here, our results add another level of complexity to *DMD* and *DMBT1* expression patterns and indicate possible involvement of circRNAs in EC pathology.

Together, our results show that altered expression of circRNAs in EC is a result of quantitative changes in the expression of specific back-spliced isoforms and a number of circular isoforms, with specific exon composition expressed by individual gene loci. However, a limitation of the current study was the relatively small sample size. Future studies should include larger samples sizes and evaluate whether circRNAs are altered in different stages of cancer development and molecular subtypes of EC. Furthermore, it will be important to investigate the mechanisms leading to perturbation of circRNA expression in EC, and the impact of differentially expressed circRNAs on EC-specific oncogenesis and cancer progression.

## MATERIALS AND METHODS

### Endometrial cancer and normal tissue samples

Institutional review board approval in accordance with Federal regulation was obtained from the University of Virginia Health System and patients were consented and enrolled in a prospective trial where endometrial samples were collected at surgery (hysterectomy) from postmenopausal women (mean body mass index of 35.3 kg/m^2^), with FIGO-defined low stage (1A–1C) and grade (1–2) type I endometrial cancer. Both cancer and adjacent non-cancerous endometrial tissue were obtained from six patients (Table 1), as previously described by Byrne *et al.* [47].

### Transcriptome sequencing

Total RNA was extracted from endometrial tissue samples using an RNeasy Mini Kit from Qiagen. RNA samples were ribosomal RNA (rRNA)-depleted and prepared for sequencing according to the Illumina TruSeqRNA sample preparation guide and then subjected to 120 base pair (bp) paired-end sequencing using the Illumina HiSeq2500.

### Identification of circRNAs in transcriptome data

A total of 563 million reads from twelve samples including six endometrial cancer samples and six patient-matched normal endometrial samples were analysed. The reads were trimmed using Trimmomatic [48] before being mapped to UCSC human genome hg38 using BWA-MEM [49], only alignments with a score of at least 19 bp were reported. BWA-MEM allows split alignment which is vital for circular RNA detection. BWA-MEM aligned reads were then processed by CIRI (downloaded from https://sourceforege.net/projects/ciri/) for circRNA detection and abundance calculation. CIRI determines circRNAs by the presence of paired chiastic clipping signals where two segments of a junction read was mapped to the reference genome in a reversed order [50]. To decrease the false-positive rate, putative circRNAs were further filtered based on the presence of GT-AG splicing sites and paired-end mapping whose signal was affirmed when the pair mate of a chiastic junction read was mapped in between the two chiastic segments.

### Determination of circRNA differential expression

Gene differential expression across two conditions was determined by edgeR, which is an R package, using the likelihood ratio test (LRT) based on generalized linear model which estimates probability distributions according to mean-variance relationship of each gene. LRT is analogous to paired *t*-test: it tests cancer vs normal differential expression in a pairwise fashion after adjusting baseline differences across the six patients [51]. Only genes with expression greater than 0.1 count per million (CPM) in at least six samples were selected for differential testing. For the total library sizes of the 12 samples in this study, 0.1 CPM corresponds to 4–6 reads. Genes with a reported FDR values less than 0.1 were considered differentially expressed.

## SUPPLEMENTARY MATERIALS FIGURES AND TABLES






