# Supplementary material for: Analysis of the circular RNA transcriptome in endometrial cancer
Source: Oncotarget. 2017 Dec 20;9(5):5786–96. doi: 10.18632/oncotarget.23534 (PMC5814174; doi:10.18632/oncotarget.23534)
Supplement: Supplementary file 1 [file oncotarget-09-5786-s001.pdf]

Analysis of the circular RNA transcriptome in endometrial cancer

SUPPLEMENTARY MATERIALS

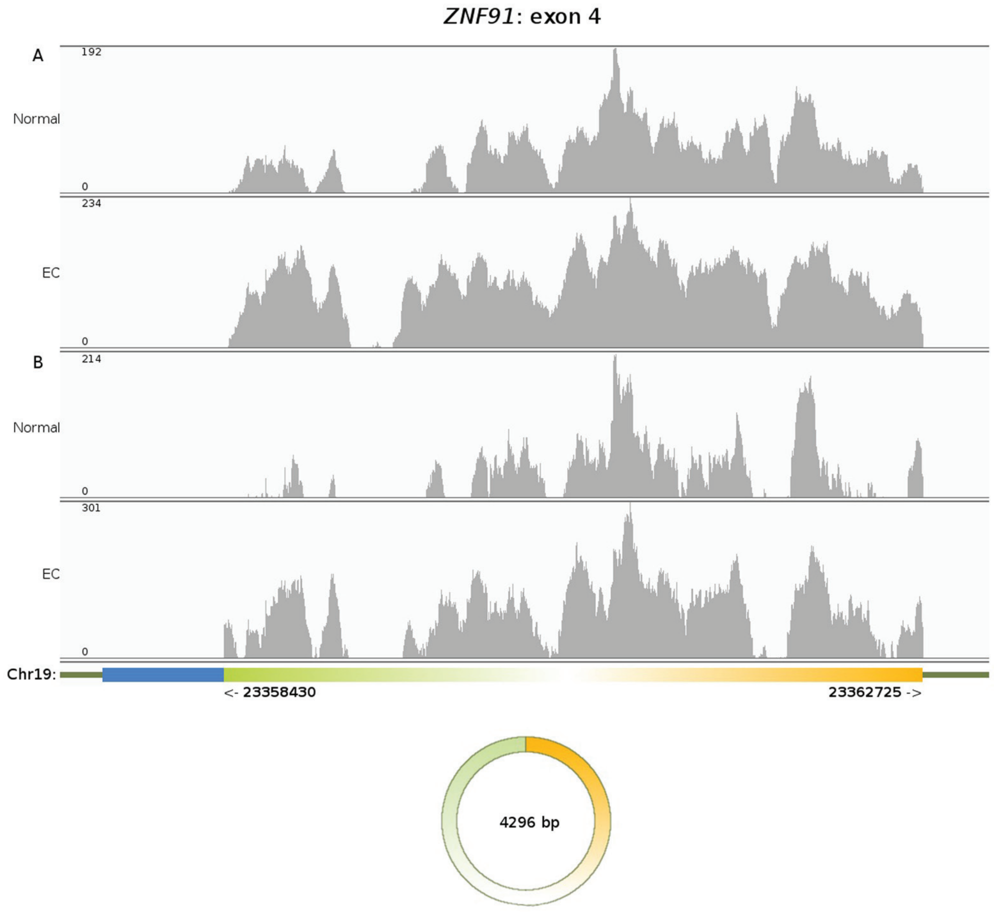

Supplementary Figure 1: Schematic presentation of exon expression and structure of Zinc Finger Protein 91 (*ZNF91*) gene for (**A**) linear and (**B**) circular transcripts. Peaks within the exon are the reads mapped to the exon. The level of expression is reflected by the height of the read peaks. Below (B), a circular RNA form of *ZNF91* is illustrated as a product of back-splicing.

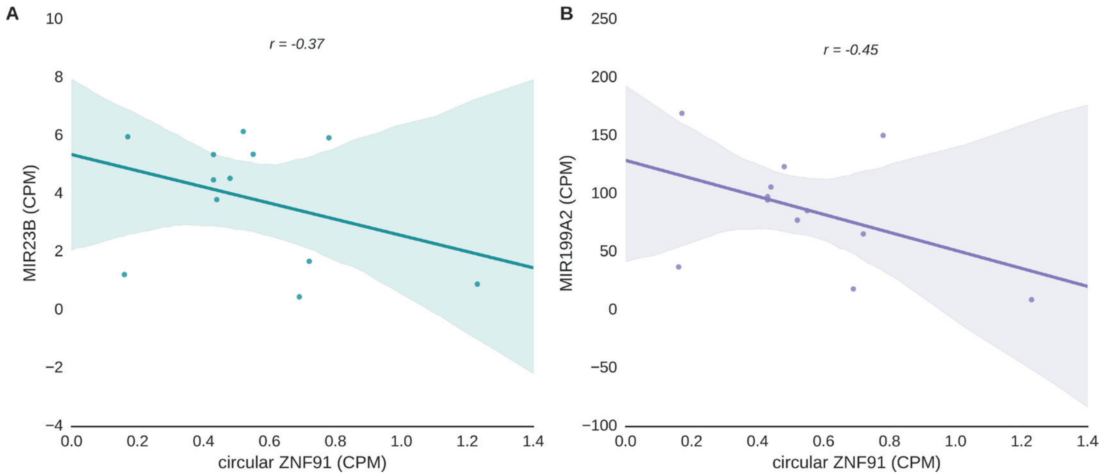

Supplementary Figure 2: Pearson's correlation coefficients between circular ZNF91 with (**A**) miR23B, and (**B**) miR199A2.

Supplementary Table 1: Genes that produced more than ten circRNA isoforms in endometrial cancer and normal tissues, respectively. See Supplementary_Table_1

Supplementary Table 2: Differentially expressed circular RNAs across endometrial cancer and normal tissues. See Supplementary_Table_2
